# An integrated microfluidic system using a micro-fluxgate and micro spiral coil for magnetic microbeads trapping and detecting

**DOI:** 10.1038/s41598-017-13389-x

**Published:** 2017-10-11

**Authors:** Xuecheng Sun, Zhu Feng, Shaotao Zhi, Chong Lei, Di Zhang, Yong Zhou

**Affiliations:** 1 Key Laboratory for Thin Film and Microfabrication of the Ministry of Education, Department of Micro/Nano Electronics, School of electronic information and electrical engineering, Shanghai Jiao Tong University, Dongchuan Road 800, Shanghai, 200240 China; 2 Center for Advanced Electronic Materials and Devices, Shanghai Jiao Tong University, Dongchuan Road 800, Shanghai, 200240 China

## Abstract

We report an innovative integrated microfluidic platform based on micro-fluxgate and micro-coils for trapping and detecting magnetic beads. A micro-spiral coil fabricated by microfabrication technology is used to trap the magnetic beads, and the micro-fluxgate is employed to detect the weak magnetic field induced by the trapped magnetic beads. The fabrication process of the magnetic bead trapping system using a micro-coil is highly compatible with that of the micro-fluxgate sensor, making fabrication of this integrated microfluidic system convenient and efficient. It is observed that the magnetic bead trapping ratio increases as the number of magnetic beads is increased with a flow rate of 5 to 16.5 μL·min^−1^. Samples spiked with different concentrations of magnetic beads can be distinguished clearly using the micro-fluxgate sensor in this microfluidic system. In this study, the results demonstrate that the microfluidic system traps and detects magnetic beads efficiently and is a promising candidate for biomarker capture and detection.

## Introduction

Since the concept of microfluidic analysis systems was introduced in 1990^1^, multiple technologies for the implementation of fluidic microsystems have been developed^2^. Microfluidic analysis systems can integrate many chemical and biological processes that tend to reduce sample/reagent consumption, as well as cost and time consumption^3^, and the systems are therefore promising for many biological and medical applications. Meanwhile, superparamagnetic beads, which possess advantages, such as physical and chemical stability, higher environmental safety and bio-compatibility, low background signal, and long and stable magnetic characteristics^4^, have triggered numerous breakthroughs in advanced biology and medicine^5–9^, such as cell manipulation^10,11^, biomarker separation and transportation^12,13^, mixing^14^, bioassay^15^ and DNA or RNA hybridization^16,17^. These applications primarily depend on the manipulation of magnetic beads by a magnetic force^18,19^ or shear-induced inertial lift force^20^. Based on the development of microfluidics and magnetic bead technology, a new “magneto-microfluidic” area of research has developed, and it primarily includes two aspects: magnetic bead manipulation and magnetic bead detection^13^. To date, several magnetic bead manipulation techniques have been reported, and most of them employ either permanent magnets or conventional electromagnets^21–23^, which are usually bulky and difficult to integrate into lab-on-a-chip systems. With the development of MEMS technology, micro-coils fabricated by microfabrication^24^, which provide a seamless bioprocess on a chip with a small volume of reagents and samples, has attracted significant attention because it offers a great selection in the choice of complementary magnetic separation method. Moreover, a number of magnetic biosensor technologies based on detecting the fringe magnetic field induced by the magnetic beads within labelling biomarkers have been developed. Successful measurements of magnetic permeability^25^, magneto-resistive sensors^26^, spin-valves^27^, superconducting quantum interference devices (SQUIDs)^28,29^, Hall sensors^30,31^ and giant magneto-impedance sensors^32^ have been reported using these devices.

Although many magnetic bead manipulation systems and magnetic bead detecting systems have been developed in recent years, the number of microfluidic systems that can accomplish both magnetic bead manipulation and detection is still notably low. The challenge of system integration results from two factors. First, common magnetic bead detection and manipulation systems too large to be suitable for application to microfluidic systems^33^. Second, the fabrication of the detection and manipulation systems are not compatible with each other, which makes system integration quite difficult. If these two barriers are conquered, the microfluidic system can offer many exciting possibilities for future developments.

Micro-fluxgate sensors are widely used to measure weak magnetic fields on account of their high sensitivity, quick response, ease of miniaturization, wide measurement range^34,35^, and integrability onto microfluidic chips^36^. Biosensor systems using micro-fluxgates are highly suitable for detecting biomarkers in a microfluidic system^37^. Our previous work has confirmed that micro-fluxgates with detection sensitivity of 90 to 100 magnetic beads are a potential candidate for biomarker detection^36,38,39^. Furthermore, the fabrication process of magnetic bead trapping systems using a micro-coil is very compatible with the micro-fluxgate structure^38^, and both can be fabricated using common microfabrication processes. On account of these advantages, we try to develop a microfluidic platform that incorporates both a micro-fluxgate and a magnetic trapping system.

In this paper, an integrated microfluidic platform was designed for magnetic bead manipulation and detection. Magnetic bead capture was implemented by the micro-coil, while the micro-fluxgate was employed for detecting the trapped magnetic beads. The relationships between the magnetic bead trap ratio and the injected current for the micro-coil, the bead flow rate and the bead concentration were investigated in detail. To the best of the authors’ knowledge, this report describes the first demonstration of a microfluidic platform using a micro-fluxgate sensor to detect magnetic beads.

## Methods and Materials

### Design and simulation of trapping system

The micro-coil is compatible with the microfabrication technology used for integrating the micro-fluxgate and is accurate in bead manipulation^40,41^; therefore, it was selected for magnetic bead trapping in this microfluidic system. According to the work of Zborowski^42^, when a magnetic field is present, a magnetic force will be exerted on the magnetic bead. Equation 1 shows the relationship between the magnetic field B and the trapping force exerted on the magnetic bead.

1 $${{\rm{F}}}_{mag}=\frac{V\Delta {\rm{\chi }}}{{\mu }_{0}}(\mathop{B\,}\limits^{\longrightarrow}\cdot \nabla )\mathop{B\,}\limits^{\longrightarrow}$$

where V is the volume of the bead, Δχ is the volumetric magnetic susceptibility difference between the particle and the surrounding buffered fluid medium, and μ_0_ is the vacuum permeability. The F_mag_ depends on the magnetic field and its gradient. In addition to the magnetic force, the bio-molecule-magnetic bead composite experiences hydrodynamic drag and gravitational forces. The hydrodynamic drag force is a consequence of the velocity difference between the magnetic particle and the liquid and, for a spherical particle with radius R, is given by^43^

2 $${\rm{F}}=-6{\rm{\pi }}{\rm{\eta }}R\cdot \vec{V}$$

with the assumption that the bio-molecule-bead composite has the approximate shape of a sphere of radius R and where η is the solution viscosity and $\vec{V}$ is the composite velocity. The effect of gravity is assumed to be negligible due to the extremely small size of the particles. The hydrodynamic drag force is the most important competing force opposing the magnetic force acting on the bead. Under conditions typical for trapping of a magnetic bead, i.e., η = 10^−3^ N·sm^−2^, R = 2.8 × 10^−6^ m and v ≈ 0.5 × 10^−4^ m·s^−1^, the drag force is F_drag_ ≈ 2.63 × 10^−12^ N. The magnetic capture force will dominate if the magnetic force is larger than 2.63 × 10^−12^ N.

The commercial magnetic particles used in this study are Magnetic beads^®^ Myone^TM^ magnetic microspheres with an average diameter of 2.8 μm, a 14% magnetic content and a magnetic susceptibility^44^ of 0.336. These data are used in the calculations along with the conditions imposed to make the magnetic force dominate other competing forces for magnetic bead capture. According to results from Ramadan^45^, ∇B^2^ should be larger than 0.206 T^2^·m^−1^ to trap magnetic beads with 2.8 μm diameter.

**Figure 1:**
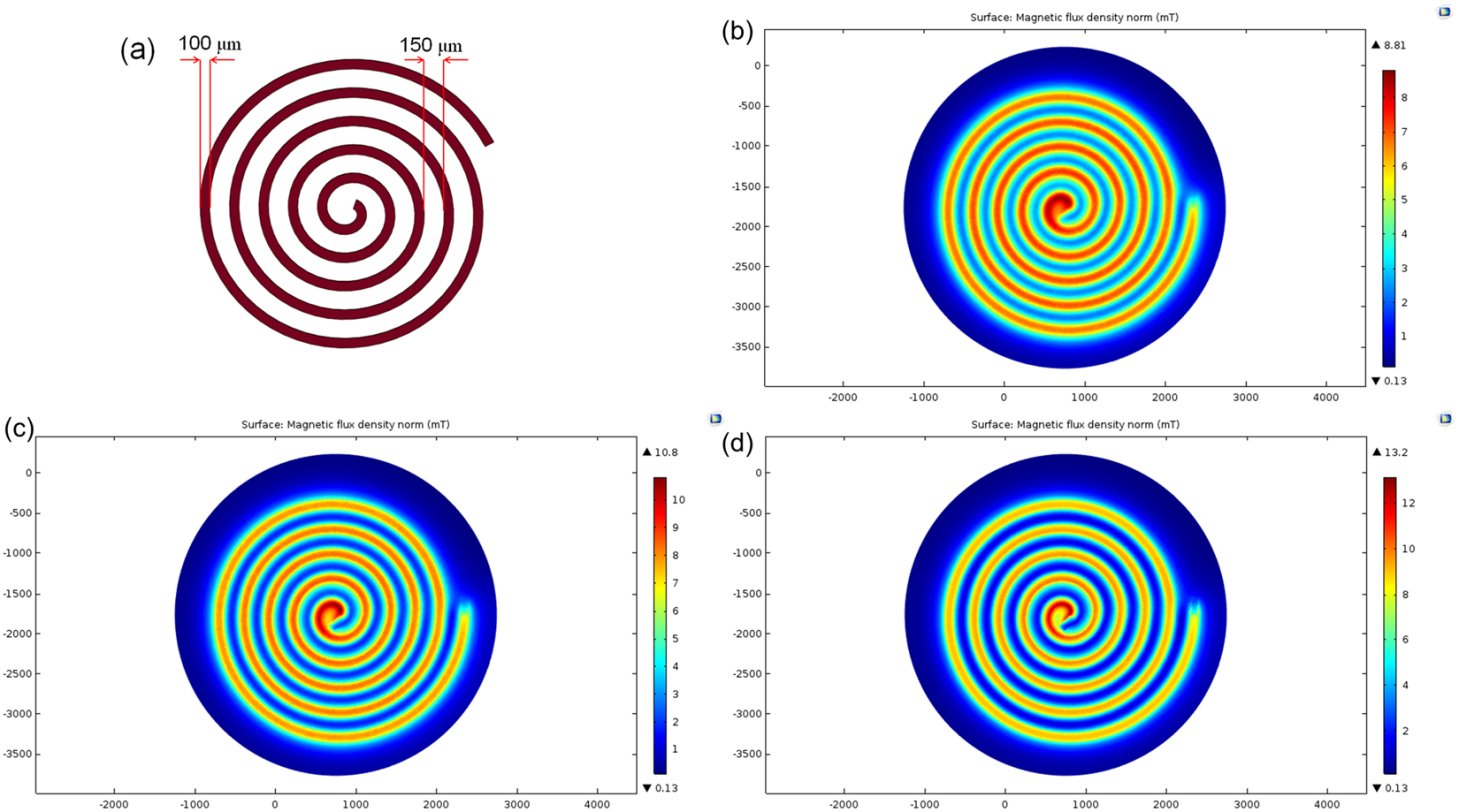
(**a**) Schematic of the micro-coils design in this work; (**b**,**c**,**d**) The simulation of micro coil with injected current was 400, 500 and 600 mA.

In this paper, we design a micro-coil with spiral geometry^24^. To prevent the magnetic bead from clustering, increase the capture area and reduce the Joule heating problem, we have designed the spiral coil with a width of 100 μm, thickness of 50 μm, and a gap of approximately 150 μm with five turns, as shown in Fig. 1a. The magnetic flux density characteristics of the spiral coil were simulated by the COMSOL software and are shown in Fig. 1b–d, for excitation currents of 400 mA, 500 mA and 600 mA, respectively.

As shown in Fig. 1c, the maximum magnetic flux density (10.8 mT) occurs at a surface 10 μm from the centre of the conductors, and the minimum magnetic flux density is achieved at the gap centre. Furthermore, ∇B ranges from 0.512 to 2.46 T^2^·m^−1^, so a large force (F = 31.2 × 10^−12^ N) can be exerted on the beads, sufficient to capture the magnetic bead from the flowing solution. The maximum magnetic flux occurs at the coil surface, and, as a result, the magnetic beads in the suspension follow the magnetic field gradient towards these maxima and are trapped at their locations.

### Micro-fluxgate fabrication

**Figure 2:**
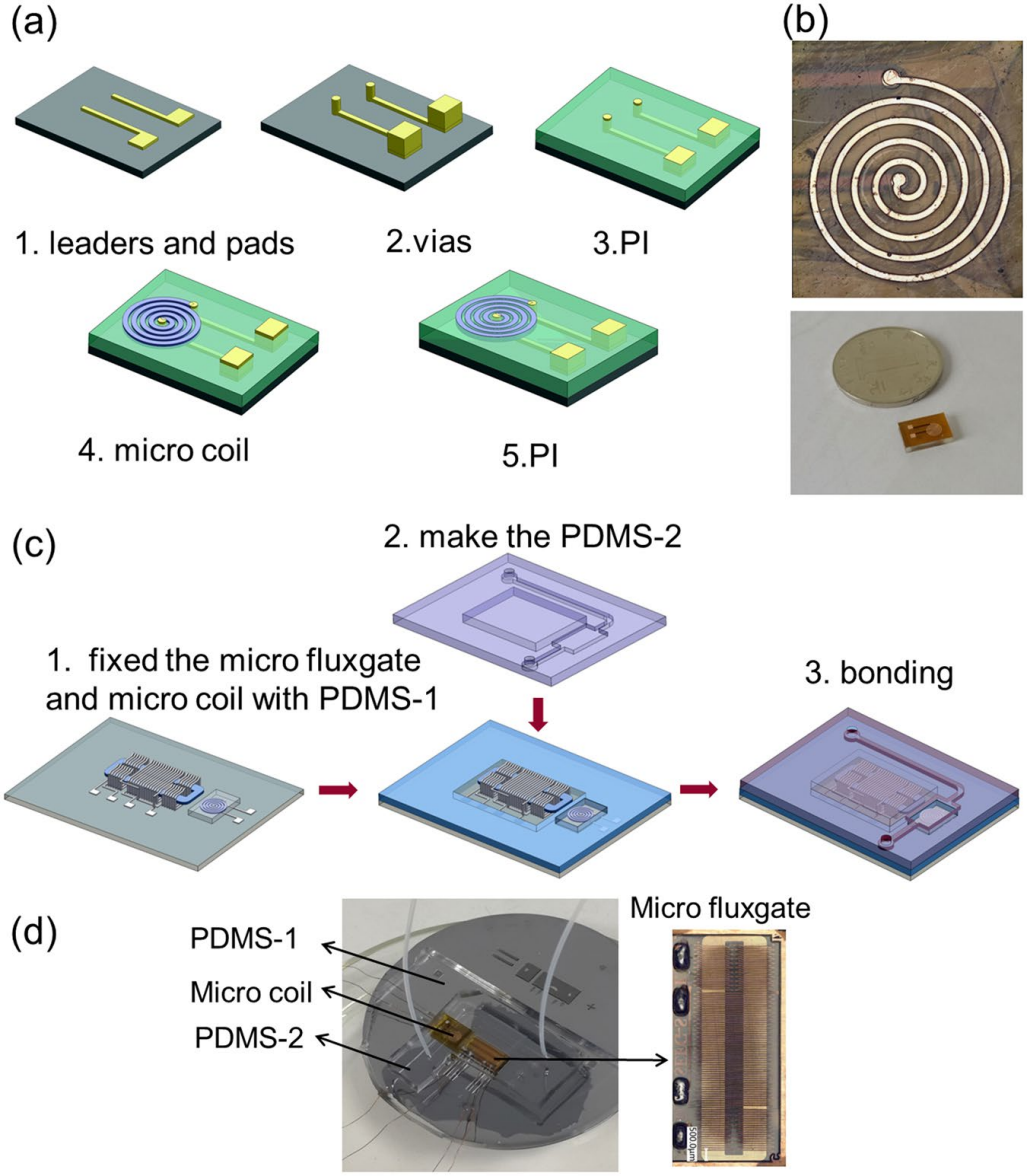
(**a**) Block diagram of the micro coil fabrication steps; (**b**) Photograph of the fabricated micro coil, (**c**) Block diagram of the microfluidic system fabrication steps; (**d**) The final microfluidic block with micro fluxgate and micro coil integrated in it.

The micro-fluxgate sensor was fabricated using standard microfabrication processes^38^. The magnetic core of the micro-fluxgate sensor is a rectangular- shaped frame made of FeNi (permalloy). The width of the magnetic core is 600 μm, and four excitation coils, each with 15 turns, are connected in serials. There is only one sensing coil, and it has 60 turns, and the widths of each coil line and each gap between the lines are 50 μm. The total area of the sensor is 10 mm × 5 mm. The fabricated micro fluxgate sensor is shown in Fig. 2, and the fabrication process is described in detail in the Electronic Supplementary Material.

### Fabrication of the micro-coil

The fabrication of micro coil is performed during the micro-fluxgate fabrication. The fabrication details are as follows. First, a seed layer (Cr/Cu) is sputtered on the substrate. Next, a photoresist mould consisting of pads and leads was made on the wafer, and the patterns were electroplated with copper. An additional photoresist mould consisting of vias and pads was made on the wafer and again electroplated with copper. After remove the photoresist and seed layer, a polyimide (PI) layer was solidified on the wafer surface for isolation and support. An additional seed layer (Cr/Cu) was sputtered onto the PI layer after polishing, and a photoresist mould of coils was made on the wafer and electroplated with copper. Again, photoresist and seed layer were removed, and a PI layer was solidified on the wafer surface for isolation and support. Finally, the PI layer was polished to make the coil surface flat and smooth. The fabricated micro-coil is shown in Fig. 2b.

### Fabrication of the microfluidic platform

**Figure 3:**
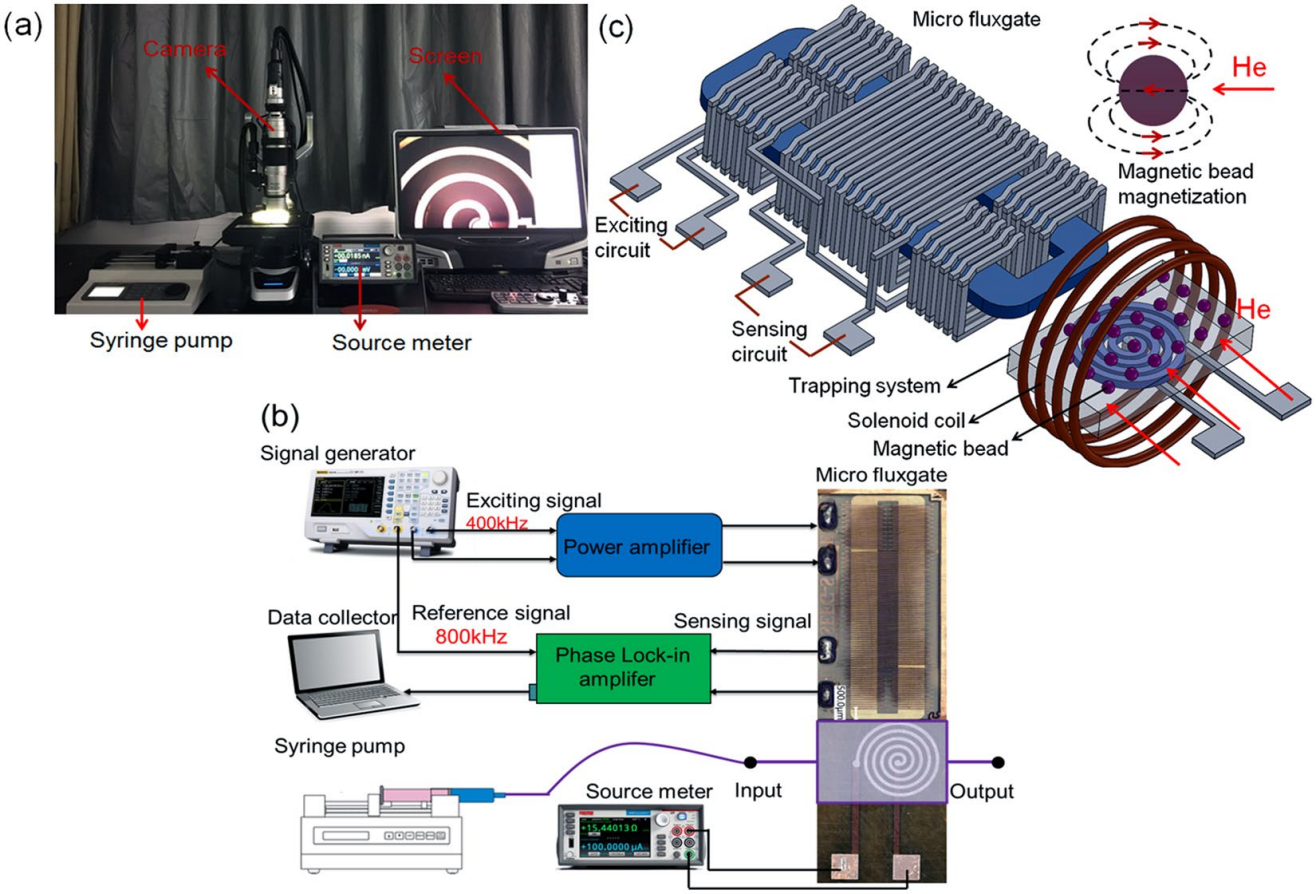
(**a**) The experiment and observation system for trapping magnetic beads by this microfluidic system; (**b**) Block diagram of the micro-fluxgate-based microfluidic detection system; (**c**) Detection discipline of the micro-fluxgate-based microfluidic system.

Polydimethylsiloxane (PDMS) material was used to fabricate micro-channels on the sensor and micro-coil to trap magnetic bead samples. The whole platform consists of two PDMS parts, namely, PDMS-1 and PDMS-2. As shown in Fig. 3, the PDMS-1 was designed to fix and cover the surfaces of the micro-coil and micro-fluxgate to construct the micro-channel via bonding with PDMS-2. The PDMS-2 was fabricated by SU-8 mould and used for shaping the micro-channel. The fabrication processes are as follows: (1) SU-8 mould: after lithography and corrosion, one layer of SU-8 is patterned with a total thickness of 100 μm, and then a Cr/Cu seed layer with 100 nm is sputtered onto the surface. (2) PDMS casting: the pre-polymer and curing agent of PDMS were mixed according to 10:1 by weight. After thermal coagulation and mould release, the appropriate structure of PDMS was obtained; (3) chip bonding: oxygen plasma treatment is applied to the surfaces of PDMS-1 and PDMS-2. The surface-treated PDMS is tightly bonded and then placed into an oven at 80 ^◦^C for 15–20 min to make a permanent bond between PDMS-1 and PDMS-2. The final, total area of the trapping section was 5 mm × 6 mm × 100 μm. The fabrication steps are shown in Fig. 2c, and the fabricated microfluidic platform is shown in Fig. 2d.

### Magnetic bead trapping and measurement system

Solutions (10 μL) of prepared magnetic bead samples (2.8 μm) with concentration of 1 μg·ml^−1^ were pumped into the fluidic chamber through Teflon tubing using a syringe pump. The magnetic devices were excited by injecting a DC current of form kthey2450. The injected current was increased until bead trapping was observed. Real-time tracking of the magnetic beads is implemented using a VHX-5000 optical microscope with CCD camera and image/video recording system. The whole experimental set-up is shown in Fig. 3a. The trapping ratios at different micro-coil conditions were measured by counting the magnetic beads directly, at both the fluid inlet and the fluid outlet, using a haemocytometer. The initial concentration of magnetic beads was fixed at 1 μg·mL^−1^ (approximately 9000~10000 beads). The trapping ratio is defined as the number of trapped beads divided by the number of injected beads.

The basic principle of magnetic bead detection is implemented by the second harmonic method. The relationship between the output second harmonic signal and the externally tested magnetic field is tested using a lock-in amplifier to read the signal value^38^. As shown in Fig. 3c, under the magnetization of the DC magnetic field, the trapped beads induce a new fringing magnetic field, which is generally monodirectional and opposite to the DC field^46^. In this case, the presence of the magnetic beads causes a decrease in the effective DC magnetic field experienced by the micro-fluxgate sensor. Thus, the magnetic beads can be detected based on the change of magnetic field measured by the micro-fluxgate sensor. The measurement system is shown in Fig. 3b.

## Results and Discussion

### Trapping the magnetic beads

**Figure 4:**
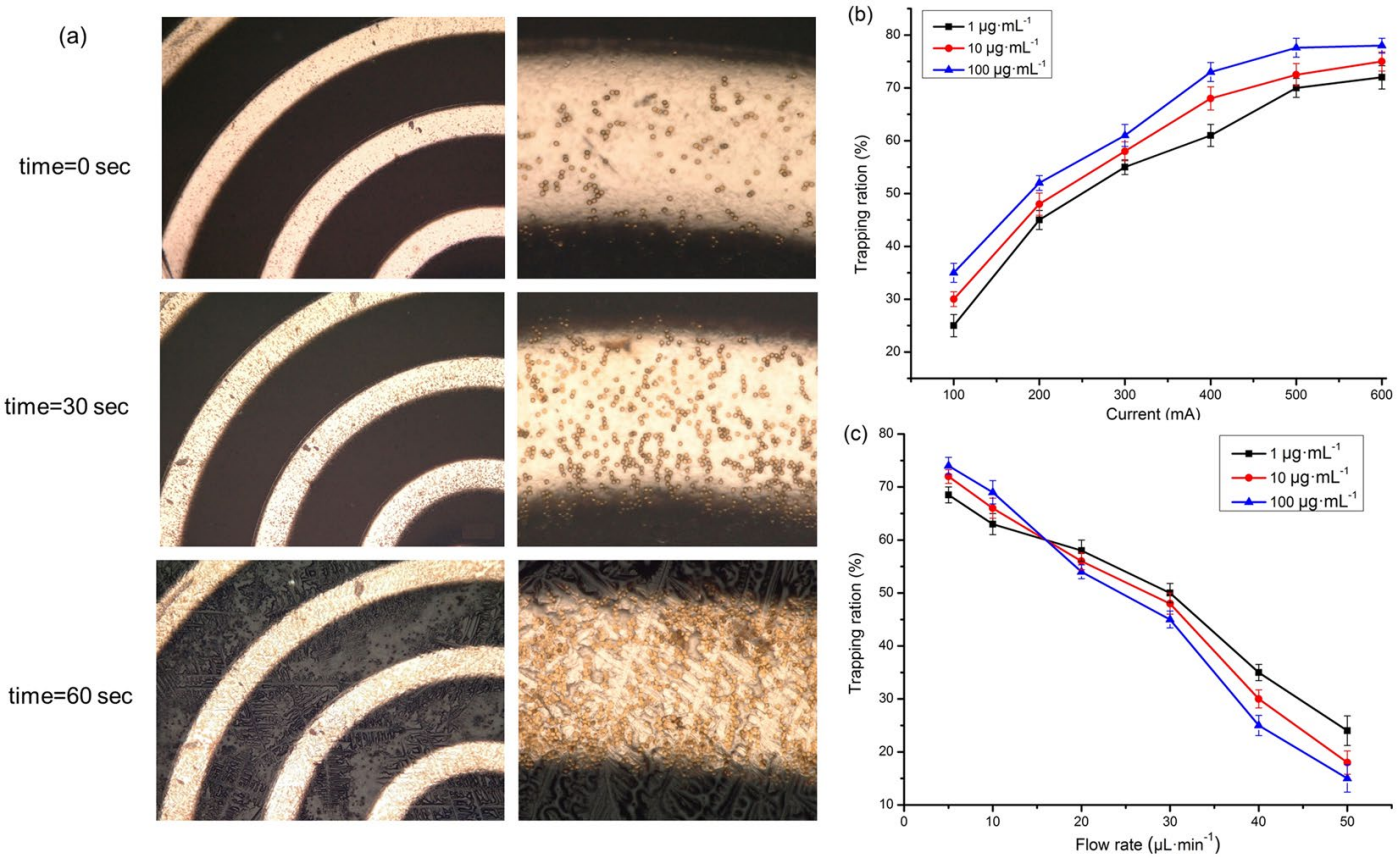
(**a**) Magnetic beads trapping sequence captured in frames taken at different time intervals on the micro coil surface; (**b**) The trapping ratio as a function of the injected dc current for the coil with the flow rate of 10 μl·min^−1^; (**c**) Trapping ratios as a function of the flow rate for micro coil at a current intensity of 500 mA.

Figure 4a shows the behaviour of magnetic beads trapped under the effect of the magnetic force generated from the micro-coil, at different time intervals and with an injected current of 500 mA. On the macroscale, the trapped beads tend to form chains oriented along the magnetic field direction due to the dipole–dipole interaction, and the number of trapped beads increases towards the inside of the spiral coil surface. The results demonstrate strong agreement between the simulated magnetic flux distribution profiles and the observed magnetic beads trapping profiles, as the locations of maxima correspond.

The trapping ratio of magnetic beads was measured under continuous flow conditions for a wide range of flow rates, and the critical flow rate, at which the micro-coil is no longer able to hold beads, was determined. As shown in Fig. 4b, the trapping ratio decreases with increasing flow rate in the range of 5 to 50 μL·min^−1^. At a flow rate of 5 μL·min^−1^, the maximum trapping ratios are 68.5%, 72.1% and 74.5%, for 1, 10 and 100 μg·mL^−1^ respectively. Moreover, the trapping ratio increases with increasing flow rate in the range of 5 μL·min^−1^ to 16.5 μL·min^−1^. This finding may be attributable to the following factors. First, with increasing number of injected beads, the interaction area between the micro-coil and the beads is increased, improving the probability of bead capture. Second, under the magnetization due to the magnetic field, the captured beads enhance the magnetic trapping gradient on the micro-coil surface. In addition, when the flow rate exceeds 16.5 μL·min^−1^, the trapping ratio decreases with decreasing concentration because most of the beads are carried away by the flow fluid.

As shown in Fig. 4c, the trapping ratio increases with increasing current and bead concentration. However, the trapping ratios tend towards saturation when the injected current is 600 mA. This may due to the heating effect resulting from the conductors^10^. The heating effect amplifies the bead vibration in the flow solution, which weakens the ability of the trapping system to capture beads. In the future, we will consider several effective methods, such as adding soft magnetic material^33^ in the micro-coil, for further decrease the injected current required for efficient bead trapping.

### Detection of magnetic beads by micro-fluxgate

**Figure 5:**
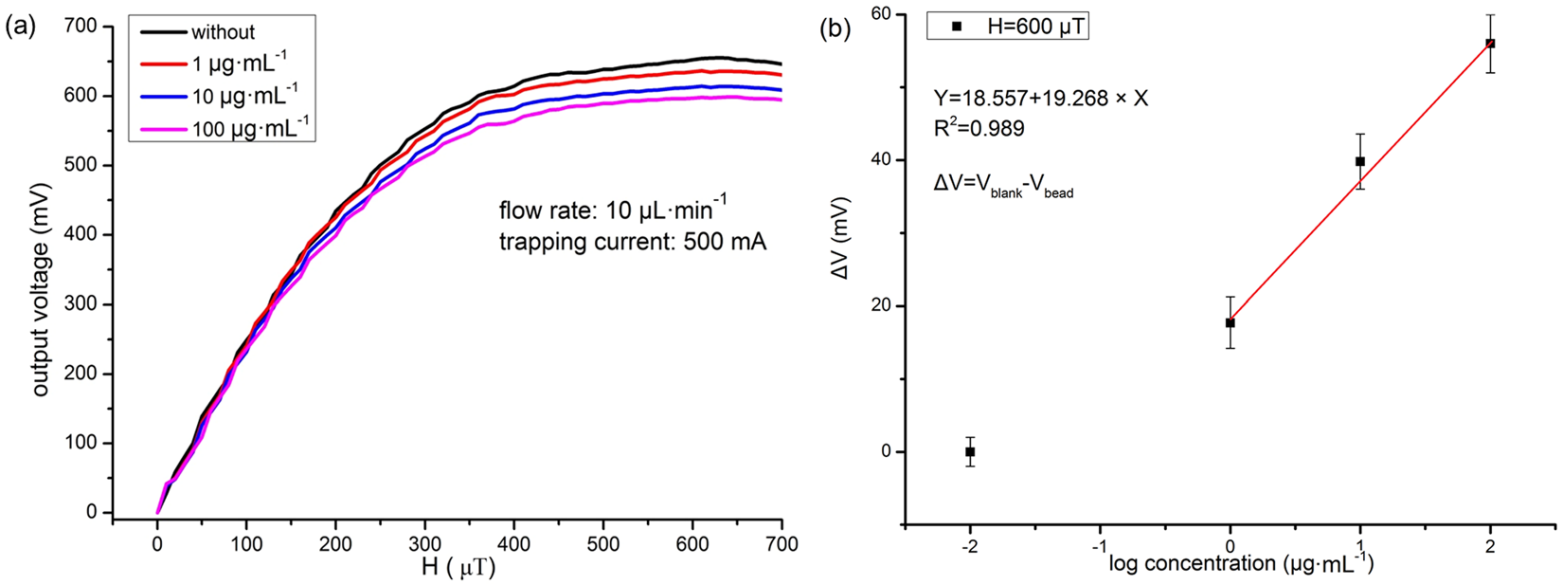
(**a**) Output signals of micro fluxgate VS trapped magnetic beads by the micro coils; (**b**) Calibration curve of measured output vs. magnetic beads concentrations.

Magnetic bead samples with concentrations of 1 μg·mL^−1^, 10 μg·mL^−1^ and 100 μg·mL^−1^ were measured. The trapping system is injected with a DC current of 500 mA, and the bead samples are pumped at a flow rate of 10 μL·min^−1^. As shown in Fig. 5a, with increasing bead concentration, the output voltage of the sensor decreases significantly. With the external magnetic field ranging from 400 μT to 700 μT, the performance of the magnetic bead samples with different concentrations can be clearly distinguished from one another. The maximum output voltages for each of the concentrations are 636.48 mV, 614.42 mV and 598.20 mV, respectively. Concentrations as low as 1 μg·mL^−1^ with a volume of 10 μL (approximately 9000–10000 × 62%) can be detected, but compared with our previous work^36^, the sensitivity is not high. There are two main reasons for this finding: First, the distance between magnetic bead trapping system and the fluxgate sensor greater than in the previous detection methods, which leads to a weaker magnetic field induced by the magnetic beads. This can, in turn, reduce the sensor’s sensitivity to the magnetic beads. Second, bead clusters pose a formidable challenge for magnetic biosensors based on the detection of beads. In our microfluidic system, magnetic beads are clustered after trapping on the micro-coil, which can even remove some of the injected current. The beads clusters can decrease the intensity of the weak magnetic field induced by the magnetic beads, thereby reducing the sensor’s ability to detect the magnetic beads^47^. We will continue to improve the design and fabrication technology of the integrated microfluidic system to overcome these problems.

Figure 5b shows the relationship between the logarithm of the concentrations of the trapped magnetic beads and the corresponding output voltage of the micro-fluxgate for an external DC magnetic field of 600 μT. The output voltage of the micro-fluxgate with no sample is V_blank_, and V_bead_ is the output voltage of the micro fluxgate with the sample. The difference between these two voltages is given by ΔV. As shown in Fig. 5b, there is a good linear relationship between the concentration and output voltage of the micro-fluxgate in the concentration range of 1 to 100 μg·mL^−1^, which can be used for further quantitative analysis. In addition, each concentration was measured 5 times, and the repeatability eR was 1.55%. Signal to noise ratio was calculated with 12 dB for detecting 1 μg·mL^−1^ sample. The performance of this sensor was almost identical when measured again after one month.

Owing to the micro-fluxgate work principle, the direction with the highest sensitivity in detecting the weak magnetic field is along the axis of the sensor^34,48^. In this regard, the detection performance in this system is different from other integrated magnetic biosensors, such as GMR^41^, GMI^49^ and spin-valve sensors^50^ that need local control over the magnetic labels and the molecules attached on the surface of the chip. In this detection model, bead capture was achieved using a small trapping system at a fixed distance from the sensor, which mitigated pollution of the sensor by chemical solutions. Therefore, the sensor surface is free to perform its functional duty of real sample detection. However, to many biosensors based immuno-capture based assays^37^, there are some challenges in real sample detection, such as repeatability, reusability. This integrated microfluidic system is also suffering the reusability problem in initial real sample detection. The challenge of reusability was in cleaning and remodifying with capture antibodies on the capture substrate (such as Au film). We are carrying on some ways to solve it, such as by adding the thickness of Au film, improving the cleaning method or employing APTES and SiO_2_ for modifying the capture antibodies^51^.

In addition, the trapping and detection systems can be integrated into the same micro-fabrication process. The sensitivity of the microfluidic platform can be enhanced by three ways: the first is by employing high performance magnetic material to improve the sensitivity of micro-fluxgate sensor, the second is by developing the microfabrication process to reduce the distance from the trapping area to the sensor^36^, the last is by optimizing geometry of micro coil to improve the diversity of the trapped magnetic beads and adding soft magnetic material in the micro coil to improve the magnetic trapping force.

## Conclusions

This paper introduced a novel microfluidic platform based on using a micro-fluxgate and a micro-coil to trap and detect magnetic beads. The results clearly demonstrate the potential for using this integrated microfluidic system for applications dealing with the manipulation and detection of biomolecules. The sensor avoids contamination from chemically reactive layers of the bio-samples because the trapping system is separate from the detection system, and the sensor therefore can maintain excellent stability. Since the fabrication of the micro-fluxgate sensor and the micro-coil are compatible with each other, the whole microfluidic system can be fabricated with one common, simplified process. In future studies, we will focus on enhancing the sensor performance and expanding the bio-sensing system to detect biomarkers.

## Electronic supplementary material

Supplementary Information
