# Author Correction: An integrated microfluidic system using a micro-fluxgate and micro spiral coil for magnetic microbeads trapping and detecting

**DOI:** 10.1038/s41598-018-31088-z

**Published:** 2018-08-21

**Authors:** Xuecheng Sun, Zhu Feng, Shaotao Zhi, Chong Lei, Di Zhang, Yong Zhou

**Affiliations:** 1 Key Laboratory for Thin Film and Microfabrication of the Ministry of Education, Department of Micro/Nano Electronics, School of electronic information and electrical engineering, Shanghai Jiao Tong University, Dongchuan Road 800, Shanghai, 200240 China; 2 Center for Advanced Electronic Materials and Devices, Shanghai Jiao Tong University, Dongchuan Road 800, Shanghai, 200240 China

Correction to: *Scientific Reports* 10.1038/s41598-017-13389-x, published online 11 October 2017

The Acknowledgements section in this Article is incomplete.

“This work is supported by The National Natural Science Foundation of China (No. 61273065), National Science and Technology Support Program (2012BAK08B05), Natural Science Foundation of Shanghai (13ZR1420800), Support fund of Shanghai Jiao Tong University (AgriX2015005), Support fund of Joint research center for advanced aerospace technology of Shanghai Academy of Spaceflight Technology-Shanghai Jiao Tong University, the Analytical and Testing Center in Shanghai Jiao Tong University, the Center for Advanced Electronic Materials and Devices in Shanghai Jiao Tong University.”

should read:

“This work is supported by The National Natural Science Foundation of China (No.61273065), National Science and Technology Support Program (2012BAK08B05), Support fund of Shanghai Jiao Tong University (AgriX2015005), Support fund of Joint research center for advanced aerospace technology of Shanghai Academy of Spaceflight Technology-Shanghai Jiao Tong University (USCAST2015-2), Support fund of aerospace technology (15GFZ-JJ02-05), the Analytical and Testing Center in Shanghai Jiao Tong University, the Center for Advanced Electronic Materials and Devices in Shanghai Jiao Tong University.”

