# Supplementary material for: An integrated microfluidic system using a micro-fluxgate and micro spiral coil for magnetic microbeads trapping and detecting
Source: Sci Rep. 2017 Oct 11;7:12967. doi: 10.1038/s41598-017-13389-x (PMC5636843; doi:10.1038/s41598-017-13389-x)
Supplement: Supplementary file 1 — Supplementary Information [file 41598_2017_13389_MOESM1_ESM.doc]

**Supplementary Information**

**An integrated microfluidic system using a micro-fluxgate and micro spiral coil for magnetic microbeads trapping and detecting**

Xuecheng Sun^1^·Zhu Feng^1^·Shaotao Zhi^1^· Chong Lei^1^*·Di Zhang^2^·Yong Zhou^1^*

^1^Key Laboratory for Thin Film and Microfabrication of the Ministry of Education,Department of Micro/Nano Electronics, School of electronic information and electrical engineering, Shanghai Jiao Tong University, Dongchuan Road 800, Shanghai 200240, China.

^2^Center for Advanced Electronic Materials and Devices, Shanghai Jiao Tong University, Dongchuan Road 800, Shanghai 200240, China

^*^ Corresponding author Email: leiqhd@sjtu.edu.cn, [yzhou@sjtu.edu.cn](mailto:yzhou@sjtu.edu.cn)

1. **Fabrication of micro fluxgate**

The procedure of fabrication started with a Cr/Cu seed layer with a thickness of 150 nm was deposited on the surface of a cleaned glass wafer. A 25μm photoresist layer was spun on the seed layer and patterned by UV lithography. Thereafter, the copper was electroplated into the photoresist mold to form the bottom segment of coils. The photoresist mold of vias was made on the wafer and copper was electroplated in it. The photoresist was removed with acetone and the seed layer was removed by reactive ion etching, and then polyimide was spun on the wafer. After solidification, the polyimide was well polished until the vias were exposed. A Cr/Cu seed layer with a thickness of 150 nm was deposited again on the surface and FeNi (permalloy) magnetic cores of 25 μm in thickness were electroplated into photoresist molds. The rest vertical vias and top parts of the coils were fabricated by lithography, electroplating and polyimide process alternately as before; the whole sensor was protected by polyimide. In addition, for connecting into the interface circuits of the sensor, 1 mm×1 mm copper electrodes with thickness of 20 μm were fabricated.


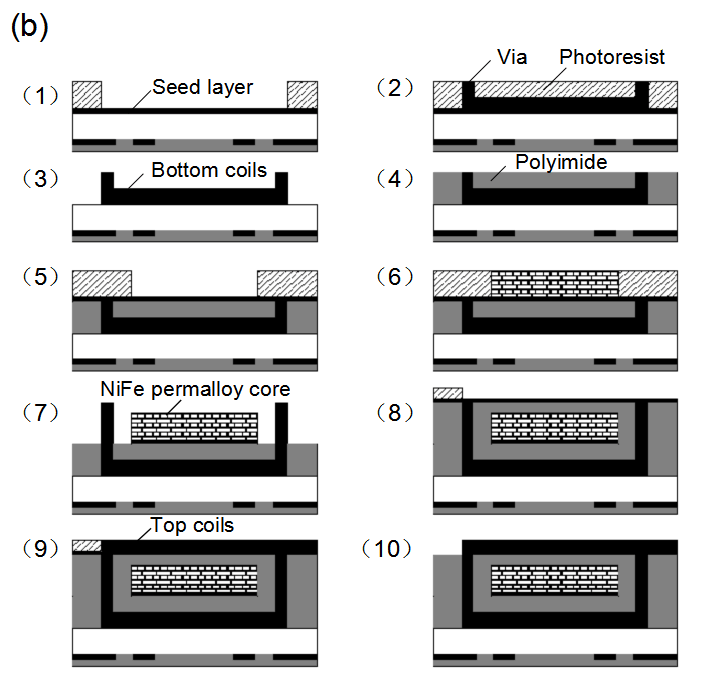


**Fig. S1. Fabrication of micro-fluxgate.**

1. **Wash experiment**

The magnetic beads which have been captured on the coil surface are easy to be washed out by fluids injected into microfluidic system without current injecting.


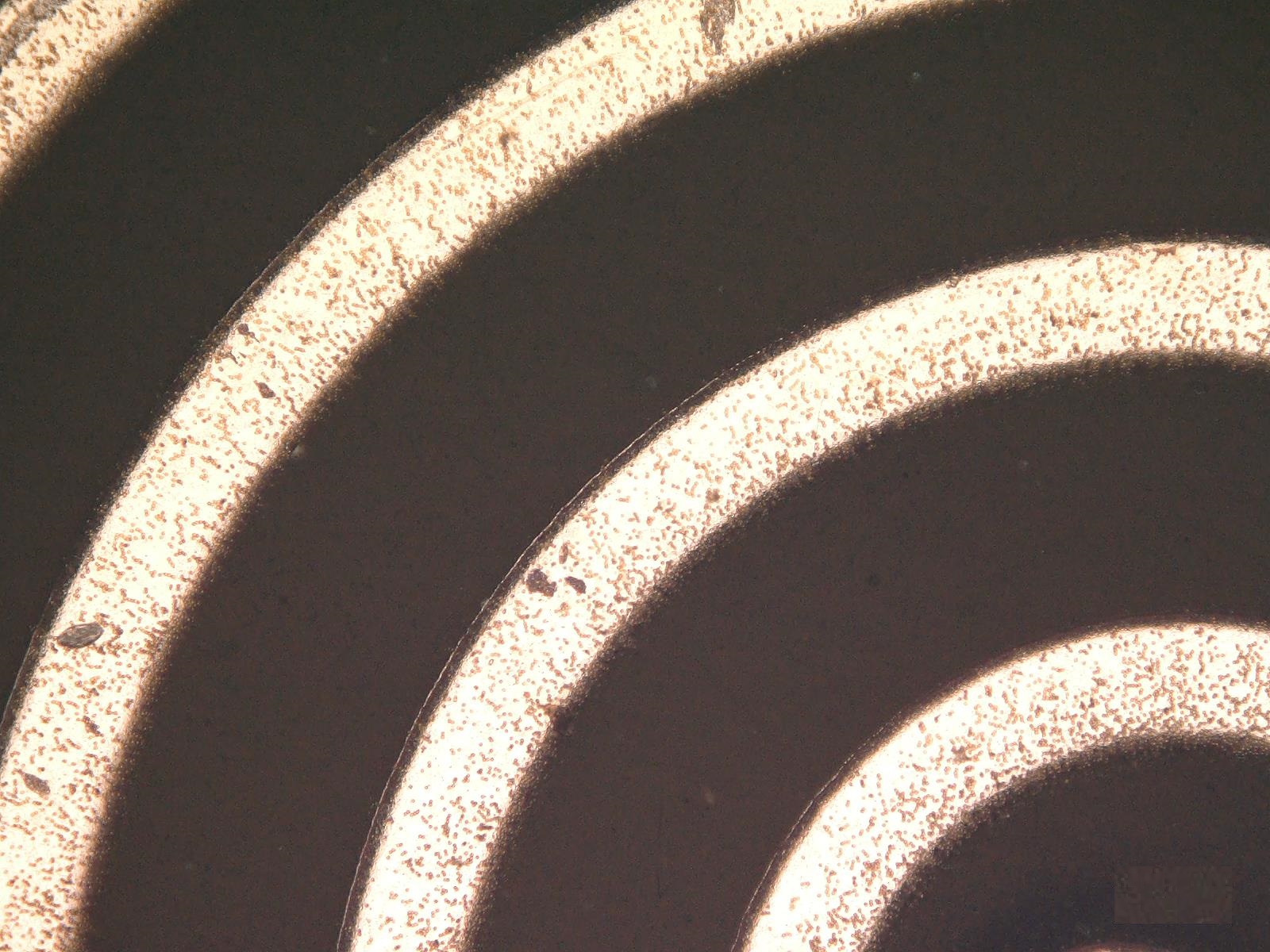

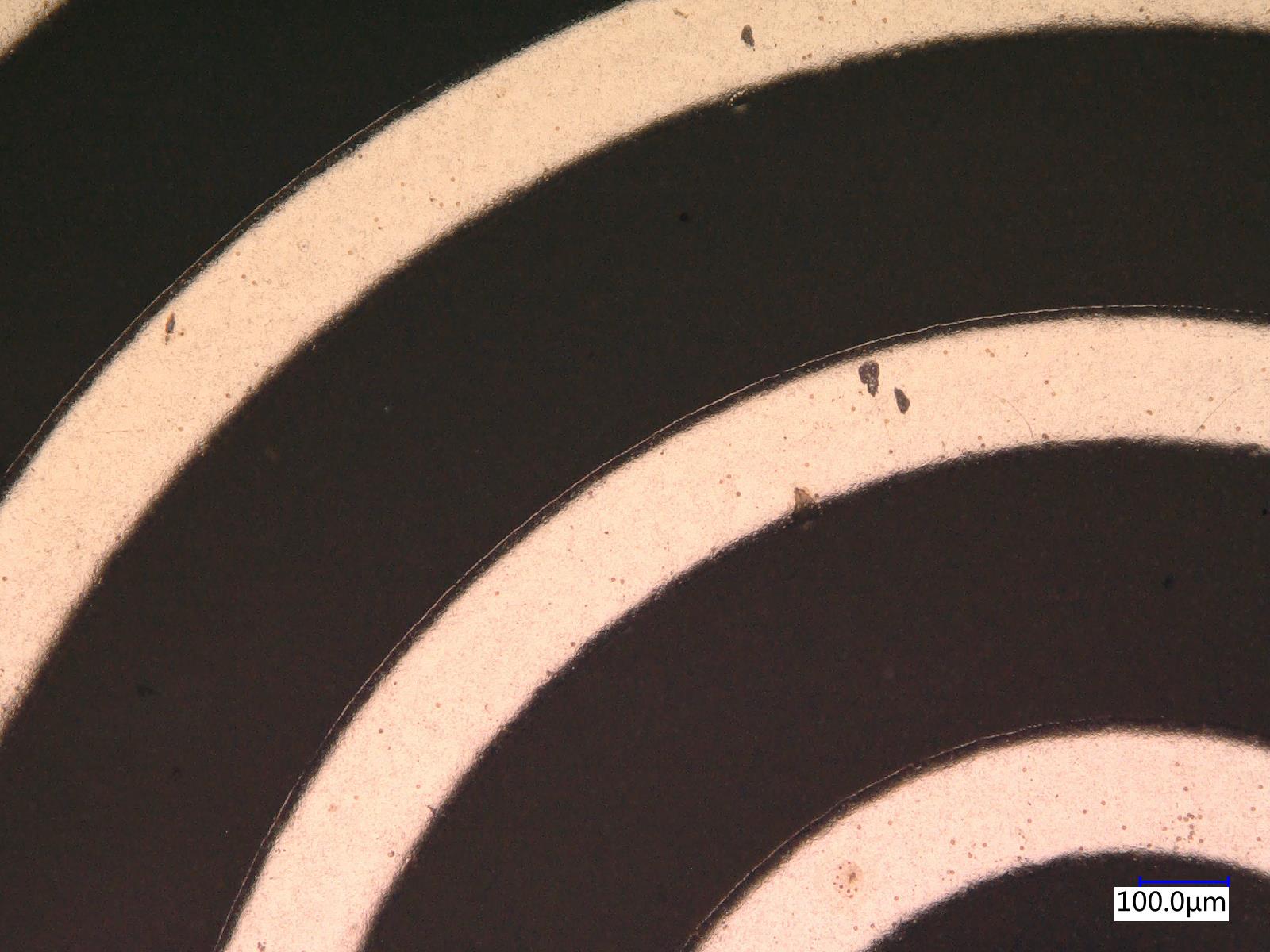


(1) (2)

**Fig. S2. The micro coil surface before (1) and after washing (2) by fluids**.
